# Most children with cancer are not enrolled on a clinical trial in Canada: a population-based study

**DOI:** 10.1186/s12885-017-3390-6

**Published:** 2017-06-05

**Authors:** Jason D. Pole, Randy Barber, Rose-Émilie Bergeron, Anne Sophie Carret, David Dix, Ketan Kulkarni, Emilie Martineau, Alicia Randall, David Stammers, Caron Strahlendorf, Douglas R. Strother, Tony H. Truong, Lillian Sung

**Affiliations:** 1 Pediatric Oncology Group of Ontario, 480 University Avenue, Suite 1014, Toronto, M5G 1V2 Canada; 2 C17 Research Council, ECHA, 11405-87 Avenue, Edmonton, T6G 1C9 Canada; 3 Montreal Children’s Hospital, 1001 Boulevard Decarie, Montreal, H4A 3J1 Canada; 4 Centre Hospitalier Universitaire Sainte-Justine, 3175 Chemin Cote Sainte-Catherine, Montreal, H1T 3C5 Canada; 5 BC Children’s Hospital, 4480 Oak Street Room B315, Vancouver, V6H 3V4 Canada; 6 IWK Health Centre, 5850/5950 University Avenue, Halifax, B3K 6R8 Canada; 7 Centre Hospitalier Universitaire de Quebec-Universite Laval, 2705 Boulevard Laurier, Quebec City, G1V 4G2 Canada; 8 Royal University Hospital, 103 Hospital Drive, Saskatoon, S7N 0W8 Canada; 9 Alberta Children’s Hospital, 2888 Shaganappi Trail N.W, Calgary, T3B 6A8 Canada; 10 The Hospital for Sick Children, 555 University Avenue, Toronto, M5G 1X8 Canada; 11 Child Health Evaluative Sciences, 686 Bay Street, Toronto, M5G 0A4 Canada

**Keywords:** Clinical trial enrollment, Children, Cancer, Canada, Population-based

## Abstract

**Background:**

Primary objective was to describe the proportion of children newly diagnosed with cancer enrolled on a therapeutic clinical trial. Secondary objectives were to describe reasons for non-enrollment and factors associated with enrollment on trials.

**Methods:**

In this retrospective cohort study, we included children newly diagnosed with cancer between 0 and 14 years of age and diagnosed from 2001 to 2012. We used data from the Cancer in Young People in Canada (CYP-C) national pediatric cancer population-based database. CYP-C captures all cases of pediatric cancer (0–14 years) diagnosed and treated at one of the 17 tertiary pediatric oncology centers in Canada. Non-enrollment was evaluated using univariate and multiple logistic regression analysis.

**Results:**

There were 9204 children with cancer included, of whom 2533 (27.5%) were enrolled on a clinical trial. The most common reasons cited for non-enrollment were lack of an available trial (52.2%) and physician choice (11.2%). In multiple regression, Asian and Arab/west Asian race were associated with lower enrollment (*P* = 0.006 and *P* = 0.032 respectively). All cancer diagnoses were more likely to be enrolled compared to astrocytoma and children with acute lymphoblastic leukemia had an almost 18-fold increased odds of enrollment compared to astrocytoma (*P* < 0.0001). Greater distance from the tertiary care center was independently associated with non-enrollment (*P* < 0.0001).

**Conclusions:**

In Canada, 27.5% of children with cancer are enrolled onto therapeutic clinical trials and lack of an available trial is the most common reason contributing to non-enrollment. Future research should better understand reasons for lack of trial availability and physician preferences to not offer trials.

## Background

There has been considerable controversy about whether enrollment on clinical trials confers a benefit to pediatric cancer patients. Several retrospective studies have concluded that patients enrolled on clinical trials have better outcomes compared with patients not enrolled on clinical trials [1]. However, as highlighted by Peppercorn, [1] these studies had important limitations regarding adjustment for potential confounders and restriction to those who would have met trial eligibility and thus, there is uncertainty about the overall benefit of trial participation. In contrast, a recent study suggested that trial participation may be associated with more infectious toxicity in children with acute myeloid leukemia [2]. It is important to emphasize there are other benefits to trial participation other than the potential for improved outcomes, such as contribution to scientific knowledge, potential to help future patients and satisfaction of knowing participants have helped others [3].

Before understanding whether patients enrolled on trials have better outcomes, it is also important to understand whether pediatric cancer patients are enrolling on trials. Failure to enroll on trials may be due to multiple factors. Possibilities include that a clinical trial for a specific diagnosis does not exist, a trial has not received regulatory or ethics approval at the institution, the institution has chosen not to activate the trial, the patient did not meet eligibility criteria, an eligible patient was not offered the trial for logistical, physician or patient-related factors or because the patient or family refused the trial [1].

The proportion of children newly diagnosed with cancer enrolled on trials is reported to range from 38% [4] to 86% [5]. According to the Children’s Oncology Group, more than 60% of young patients with cancer are enrolled in trials [6]. However, there is confusion about whether these estimates reflect all children who present with cancer, or just those who are offered trials. For National Cancer Institute-sponsored trials, Canadian institutions have additional regulatory hurdles compared to US institutions. After these trials are approved by the relevant US agencies (such as the Clinical Trials Evaluation Program and the Central Institutional Review Board), they subsequently must be approved by Health Canada and local Research Ethics Boards before Canadian sites can activate them. It is uncertain whether lack of trial availability has an important impact on the proportion of non-enrolling pediatric cancer patients in Canada. It is important to note that clinical trials include both therapeutic trials directed at cancer treatment and supportive care trials focused on reducing toxicity or improving quality of life. This study is focused on enrollment on therapeutic trials.

In order to address the question of enrollment to therapeutic clinical trials, we used the Cancer in Young People in Canada (CYP-C) database, a national pediatric cancer population-based database. The primary objective was to describe the proportion of children newly diagnosed with cancer enrolled on a therapeutic clinical trial. Secondary objectives were to describe reasons for non-enrollment and factors associated with enrollment on trials.

## Methods

### Population of interest and sampling methods

*Eligibility Criteria:* (1) Children with newly diagnosed cancer 0 to 14 years of age at diagnosis; (2) Diagnosed between January 1, 2001 and December 31, 2012; (3) Diagnosis and treatment at one of the 17 pediatric oncology centers in Canada and entered into CYP-C; and (4) Diagnosis included in the International Classification of Childhood Cancer (ICCC), third edition [7]. Diagnoses eligible for ICCC are those with malignant behavior and non-malignant intracranial and intraspinal tumors [7]. We excluded patients in which it was unknown whether they were enrolled on a clinical trial for the treatment of the initial cancer diagnosis.

### Data source

The data source was CYP-C. CYP-C is a population-based registry that captures all cases of pediatric cancer diagnosed and treated at one of the 17 tertiary pediatric oncology centers in Canada. These 17 centers provide virtually all care for children with cancer <15 years of age.

CYP-C is a publicly available dataset; application for data utilization can be submitted through the C^17^ Council website (www.c17.ca/index.php?cID=70). Patients diagnosed between 0 and 14 years of age since 2001 are included and are followed for 5 years after the first or any subsequent malignancy eligible for inclusion in CYP-C. For centers in Ontario (*n* = 5), data are transferred to CYP-C from the Pediatric Oncology Group of Ontario (POGO) Networked Information System (POGONIS), a provincial population-based registry that includes similar, but not identical data elements to CYP-C. POGONIS captures 96–98% of children 0–14 years of age diagnosed with cancer as compared with the Ontario Cancer Registry [8]. For centers outside of Ontario (*n* = 12), data are entered directly into CYP-C. Elements captured by CYP-C include demographic variables (sex, date of birth, postal code and race), diagnostic details, times to diagnosis and treatment, details of treatment plans and outcomes such as relapse, second malignancy and death.

Enrollment on a therapeutic trial is explicitly collected by both databases. At each center, identification of patients who are enrolled on a clinical trial is abstracted from the medical records. Only enrollment on a trial at the initial diagnosis was used for this analysis and not later during the course of therapy.

Achieving high quality data has been an objective of the program since inception and the following have been implemented to maximize data accuracy. A community of practice composed of each site’s data manager was established. The group meets monthly by teleconference to discuss difficult cases and face-to-face annually to further solidify training. Site audits to each site outside of Ontario have been conducted and more specifically, the section on enrollment on trials was included in the audit. Data quality issues have not been identified for this element (personal communication, Randy Barber, April 23, 2017).

For the secondary objective, related to describing reasons for non-enrollment, the CYP-C database consistently collected the stated reasons for non-enrollment using a standardized list throughout the study period. Reasons for failure to enroll on a clinical trial were categorized as follows: (1) Language barrier, trial not offered; (2) No available trial at the time; (3) Not eligible for any available trial; (4) Physician choice; (5) Refused therapy; (6) Refused to participate in proposed trial; (7) Other, specify; and (8) Information not available. Only one reason could be selected. Free text options could be indicated if the “Other” category was chosen. If not eligible was chosen, it was implied that a trial was available for that patient based upon diagnosis and age. In contrast, the POGONIS database collected reasons for non-enrollment using a standardized list only for patients diagnosed after 2010. Thus, only data from the 12 non-Ontario centers was included for the description of reasons for non-enrollment.

### Statistical plan

Primary analyses were descriptive. Categorical variables were compared between those enrolled and not enrolled on trials using Chi square tests.

In order to describe factors associated with enrollment, the following variables were examined: age at diagnosis (<1, 1–4, 5–9 and 10–14), sex, race (as collected by CYP-C), diagnosis era (< 2007 and ≥2007; midpoint of study), and sociodemographic factors. Sociodemographic factors were determined by using postal codes at diagnosis to obtain distance to the nearest tertiary care pediatric cancer center and area-level socioeconomic status by linking to census data. Full 6 digit postal codes were available for all provinces except for British Columbia in which only 3 digit postal codes were available. We used the Statistics Canada Postal Code Conversion File software (PCCF+, Version 4J) and linked the postal code at the time of diagnosis to a 2001 census dissemination area. Dissemination areas are the smallest unit of geography defined by Statistics Canada and include between 400 and 700 persons. Using the 2001 census, income quintiles were determined that adjust for household size and regional differences [9].

Factors associated with enrollment on trial were evaluated using logistic regression analysis and associations were illustrated using odds ratios (OR) with associated 95% confidence intervals (CIs). Univariate and multiple logistic regression analyses were conducted where multiple regression included all evaluated factors in the model. In order to remove the impact of lack of trial availability, we also conducted a subgroup analysis in which we removed patients in which the reason for non-enrollment was stated to be “No available trial”.

Statistical significance was defined as *P* value <0.05. Statistical analysis was conducted using the SAS statistical program (SAS-PC, version 9.4; SAS Institute Inc., Cary, North Carolina).

## Results

**Fig. 1:**
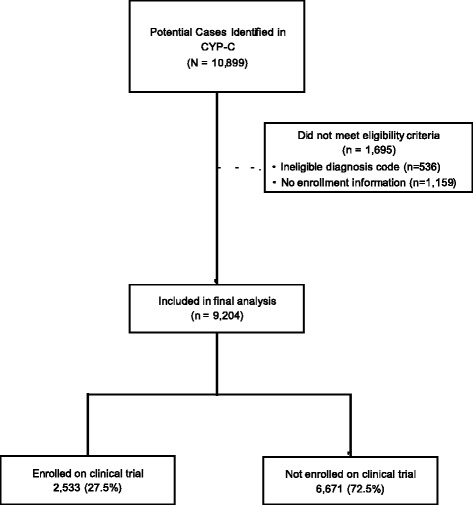
Flow diagram of case identification and selection

Of 10,899 patients identified in CYP-C, 1695 were excluded leaving 9204 that were included in the final analysis. Figure 1 illustrates the flow diagram of patient identification and selection and reasons for exclusion. Table 1 illustrates the demographic characteristics of the study cohort. Among this cohort, 2533 (27.5%) were enrolled on a therapeutic clinical trial for the initial cancer diagnosis. The number of children enrolled on a clinical trial from POGO and non-POGO centers were 867/3447 (25.2%) and 1666/5757 (28.9%) respectively (*P* < 0.0001) (data not shown). In comparing those with and without enrollment information available, there was no difference in gender (*P* = 0.581), However, there was a significant difference in diagnosis (*P* < 0.0001) and age group (*P* < 0.0001).

**Table 1:** Demographics of the Study Population

Characteristics	*N* = 9204
Demographic Features	
Age at Diagnosis	
< 1 years	991 (10.8%)
1–4 years	3355 (36.5%)
5–9 years	2324 (25.2%)
10–14 years	2534 (27.5%)
Male Sex	4961 (53.9%)
Race	
White	6169 (67.0%)
Asian	909 (9.9%)
Arab/West Asian	179 (1.9%)
Aboriginal	214 (2.3%)
Black	239 (2.6%)
Latin American	105 (1.1%)
Other	169 (1.8%)
Unknown	1220 (13.3%)
Diagnostic Era	
< 2007	4608 (50.1%)
≥ 2007	4596 (49.9%)
Diagnosis	
Leukemia	2699 (29.3%)
Acute lymphoblastic leukemia	2138 (23.2%)
Acute myeloid leukemia	395 (4.3%)
Lymphoma	962 (10.5%)
Hodgkin lymphoma	400 (4.3%)
Non-Hodgkin lymphoma excluding Burkitt’s lymphoma	340 (3.7%)
Burkitt’s lymphoma	162 (1.8%)
Solid Tumors	3187 (34.6%)
Ewing sarcoma	164 (1.8%)
Hepatoblastoma	139 (1.5%)
Neuroblastoma and ganglioneuroblastoma	734 (8.0%)
Osteosarcoma	207 (2.2%)
Rhabdomyosarcoma	273 (3.0%)
Wilms tumor	424 (4.6%)
Central Nervous System Tumors	2307 (25.1%)
Astrocytoma	982 (10.7%)
Ependymoma	197 (2.1%)
Medulloblastoma	172 (1.9%)
Socioeconomic Factors	
Median Km to Nearest Tertiary Care Center (Interquartile Range)	30.0 (12.2 to 112.4)
Income quintile	
1 (lowest)	1732 (18.8%)
2	1759 (19.1%)
3	1814 (19.7%)
4	1843 (20.0%)
5 (highest)	1893 (20.6%)
Missing	163 (1.8%)

Table 2 illustrates the reasons for non-enrollment collected from the 12 non-Ontario institutions. The most common known reasons for non-enrollment were: “No available trial”, 52.2%; “Physician choice”, 11.2%; and “Not eligible for any trial”, 6.5%. Only 3.7% of patients were not enrolled on a clinical trial because of refusal to participate. The number not enrolled because of lack of trial availability was similar before and after January 1, 2007 (52.4% versus 52.0%). The reason for non-enrollment was not found in the patient chart and was cited as unknown in 23.5% of cases. Overall, reasons for non-enrollment between the two time periods were significantly different (*P* = 0.007) by Chi square test. When focusing on the proportion with a known reason for non-enrollment, 2105/3084 (68.3%) were due to no available trial.

**Table 2:** Reasons for Non-Enrollment on Trials in 12 Non-Ontario Institutions^a^

	Total	< 2007	≥ 2007
	*N* = 4033	*N* = 2007	*N* = 2026
Language Barrier, Trial Not Offered	22 (0.5%)	13 (0.6%)	9 (0.4%)
No Available Trial	2105 (52.2%)	1051 (52.4%)	1054 (52.0%)
Not Eligible for Any Trial	261 (6.5%)	136 (6.8%)	125 (6.2%)
Physician Choice	452 (11.2%)	189 (9.4%)	263 (13.0%)
Refused Therapy	20 (0.5%)	12 (0.6%)	8 (0.4%)
Refused to Participate in Proposed Trial	151 (3.7%)	67 (3.3%)	84 (4.1%)
Other	73 (1.8%)	36 (1.8%)	37 (1.8%)
Unknown	949 (23.5%)	503 (25.1%)	446 (22.0%)

^a^In 58 cases, treatment plan was not available and thus, reason for non-enrollment was not required

Table 3 illustrates the proportion who were enrolled on a trial by underlying oncology diagnosis and illustrates that enrollment rates were highest for patients with acute lymphoblastic leukemia (ALL) (48.8%) and lowest for patients with astrocytoma (5.3%). The proportion enrolled on a trial significantly increased after January 1, 2007 for those with ALL, acute myeloid leukemia, and Wilms tumor. However, the proportion enrolled significantly decreased for those with non-Hodgkin lymphoma, other lymphomas, Ewing sarcoma, neuroblastoma and ganglioneuroblastoma, ependymoma and other central nervous system tumors.

**Table 3:** Proportion Enrolled on Trial by Diagnosis

	Total	< 2007	≥ 2007	*P* Value*
		*N* = 4608	*N* = 4596	
Leukemia	1199/2699 (44.4%)	547/1362 (40.2%)	652/1337 (48.8%)	
Acute lymphoblastic leukemia	1043/2138 (48.8%)	484/1075 (45.0%)	559/1063 (52.6%)	0.0006
Acute myeloid leukemia	138/395 (34.9%)	50/192 (26.0%)	88/203 (43.3%)	0.0005
Other leukemias	18/166 (10.8%)	13/95 (13.7%)	5/71 (7.0%)	0.267
Lymphoma	270/962 (28.1%)	167/504 (33.1%)	103/458 (22.5%)	
Hodgkin lymphoma	130/400 (32.5%)	61/200 (30.5%)	69/200 (34.5%)	0.455
Non-Hodgkin lymphoma excluding Burkitt’s lymphoma	90/340 (26.5%)	64/180 (35.6%)	26/160 (16.3%)	<0.0001
Burkitt’s lymphoma	37/162 (22.8%)	31/91 (34.1%)	6/71 (8.5%)	0.0002
Other lymphomas	13/60 (21.7%)	11/33 (33.3%)	2/27 (7.4%)	0.035
Solid Tumors	827/3187 (25.9%)	435/1556 (28.0%)	392/1631 (24.0%)	
Ewing sarcoma	63/164 (38.4%)	39/82 (47.6%)	24/82 (29.3%)	0.025
Hepatoblastoma	22/139 (15.8%)	15/70 (21.4%)	7/69 (10.1%)	0.112
Neuroblastoma and ganglioneuroblastoma	237/734 (32.3%)	140/363 (38.6%)	97/371 (26.1%)	0.0004
Osteosarcoma	78/207 (37.7%)	35/98 (35.7%)	43/109 (39.4%)	0.682
Rhabdomyosarcoma	121/273 (44.3%)	68/135 (50.4%)	53/138 (38.4%)	0.062
Wilms tumor	160/424 (37.7%)	66/204 (32.4%)	94/220 (42.7%)	0.036
Other solid tumors	146/1246 (11.7%)	72/604 (11.9%)	74/642 (11.5%)	0.898
Central Nervous System Tumors	237/2307 (10.3%)	134/1167 (11.5%)	103/1140 (9.0%)	
Astrocytoma	52/982 (5.3%)	26/508 (5.1%)	26/474 (5.5%)	0.909
Ependymoma	38/197 (19.3%)	24/93 (25.8%)	14/104 (13.5%)	0.044
Medulloblastoma	36/172 (20.9%)	15/88 (17.0%)	21/84 (25.0%)	0.274
Other central nervous system tumors	111/956 (11.6%)	69/478 (14.4%)	42/478 (8.8%)	0.009

*P values by Chi square test

In univariate analysis, factors significantly associated with enrollment on trial were age at diagnosis, race, underlying diagnosis and distance to the closest tertiary care center (Table 4). In the evaluation of diagnosis, astrocytoma was used as the reference category as these patients had the lowest rate of enrollment. In the multiple regression analysis with all factors included, age was no longer associated with enrollment (*P* = 0.450). Conversely, Asian and Arab/west Asian race remained significantly associated with lower enrollment on trials with OR 0.8 (95% CI 0.6 to 0.9; *P* = 0.006) and 0.7 (0.4 to 0.96; *P* = 0.032) respectively. In the adjusted model, all evaluated diagnoses were more likely to be enrolled on trials in comparison to children with astrocytoma and more specifically, children with ALL had an almost 18 fold increased odds of trial enrollment compared to children with astrocytoma (*P* < 0.0001). Shorter distance to the nearest tertiary care center remained independently associated with higher enrollment on trials in this multiple regression analysis. A new multiple regression model was then constructed and included site as a covariate. In this analysis, ethnicity (*P* = 0.0017) and diagnosis (*P* < 0.0001) remained significantly associated with enrollment.

**Table 4:** Univariate and Multiple Logistic Regression Evaluating Factors Associated with Enrollment

Characteristics	Enrolled *N* = 2533	Not Enrolled *N* = 6671	Percent Enrolled	Univariate Regression Analysis		Multiple Regression Analysis	
				OR (95% CI)	*P* Value*	OR (95% CI)	*P* Value*
Demographic Features							
Age at Diagnosis					<0.0001		0.450
< 1 years	216 (8.5%)	775 (11.6%)	21.8	REF		REF	
1–4 years	1078 (42.6%)	2277 (34.1%)	32.1	1.7 (1.4 to 2.0)	<0.0001	1.1 (0.9 to 1.4)	0.213
5–9 years	620 (24.5%)	1704 (25.5%)	26.7	1.3 (1.1 to 1.6)	0.003	1.0 (0.8 to 1.3)	0.711
10–14 years	619 (24.4%)	1915 (28.7%)	24.4	1.2 (1.0 to 1.4)	0.099	1.0 (0.8 to 1.3)	0.714
Sex							
Male	1405 (55.5%)	3556 (53.3%)	28.3	1.1 (1.0 to 1.2)	0.064	1.0 (0.9 to 1.1)	0.660
Female	1128 (44.5%)	3115 (46.7%)	26.6	REF			
Race					0.0003		0.033
White	1793 (70.8%)	4376 (65.6%)	29.1	REF		REF	
Asian	218 (8.6%)	691 (10.4%)	24.0	0.8 (0.7 to 0.9)	0.002	0.8 (0.6 to 0.9)	0.006
Arab/West Asian	37 (1.5%)	142 (2.1%)	20.7	0.6 (0.4 to 0.9)	0.015	0.7 (0.4 to 0.96)	0.032
Aboriginal	53 (2.1%)	161 (2.4%)	24.8	0.8 (0.6 to 1.1)	0.174	0.8 (0.5 to 1.1)	0.189
Black	66 (2.6%)	173 (2.6%)	27.6	0.9 (0.7 to 1.2)	0.628	1.1 (0.8 to 1.5)	0.654
Latin American	25 (1.0%)	80 (1.2%)	23.8	0.8 (0.5 to 1.2)	0.241	0.8 (0.5 to 1.4)	0.485
Other	50 (2.0%)	119 (1.8%)	29.6	1.0 (0.7 to 1.4)	0.883	1.1 (0.7 to 1.6)	0.697
Unknown	291 (11.5%)	929 (13.9%)	23.9	0.8 (0.7 to 0.9)	0.0002	1.1 (0.9 to 1.2)	0.488
Diagnostic Era							
< 2007	1283 (50.7%)	3325 (49.8%)	27.8	1.0 (0.9 to 1.1)	0.488	1.0 (0.9 to 1.1)	0.966
≥ 2007	1250 (49.3%)	3346 (50.2%)	27.2	REF			
Diagnosis					<0.0001		<0.0001
Leukemia							
Acute lymphoblastic leukemia	1043 (41.2%)	1095 (16.4%)	48.8	17.0 (12.7 to 22.8)	<0.0001	17.7 (12.9 to 24.4)	<0.0001
Acute myeloid leukemia	138 (5.4%)	257 (3.9%)	34.9	9.6 (6.8 to 13.6)	<0.0001	9.0 (6.2 to 13.3)	<0.0001
Other leukemia	18 (0.7%)	148 (2.2%)	10.8	2.2 (1.2 to 3.8)	0.007	2.2 (1.2 to 4.2)	0.012
Lymphoma							
Hodgkin lymphoma	130 (5.1%)	270 (4.0%)	32.5	8.6 (6.1 to 12.2)	<0.0001	10.2 (7.0 to 15.0)	<0.0001
Non-Hodgkin lymphoma excluding Burkett’s lymphoma	90 (3.6%)	250 (3.7%)	26.5	6.4 (4.5 to 9.3)	<0.0001	7.4 (4.9 to 11.0)	<0.0001
Burkitt’s lymphoma	37 (1.5%)	125 (1.9%)	22.8	5.3 (3.3 to 8.4)	<0.0001	6.0 (3.7 to 9.8)	<0.0001
Other lymphoma	13 (0.5%)	47 (0.7%)	21.7	4.9 (2.5 to 9.7)	<0.0001	6.7 (3.3 to 13.6)	<0.0001
Solid Tumors							
Ewing sarcoma	63 (2.5%)	101 (1.5%)	38.4	11.2 (7.3 to 17.0)	<0.0001	13.6 (8.6 to 21.3)	<0.0001
Hepatoblastoma	22 (0.9%)	117 (1.8%)	15.8	3.4 (2.0 to 5.7)	<0.0001	3.9 (2.2 to 6.8)	<0.0001
Neuroblastoma and ganglioneuroblastoma	237 (9.4%)	497 (7.5%)	32.3	8.5 (6.2 to 11.7)	<0.0001	9.3 (6.5 to 13.2)	<0.0001
Osteosarcoma	78 (3.1%)	129 (1.9%)	37.7	10.8 (7.3 to 16.1)	<0.0001	12.7 (8.2 to 19.8)	<0.0001
Rhabdomyosarcoma	121 (4.8%)	152 (2.3%)	44.3	14.2 (9.9 to 20.6)	<0.0001	16.3 (11.0 to 24.4)	<0.0001
Wilms tumor	160 (6.3%)	264 (4.0%)	37.7	10.8 (7.7 to 15.3)	<0.0001	11.8 (8.2 to 17.2)	<0.0001
Other solid tumor	146 (5.8%)	1100 (16.5%)	11.7	2.4 (1.7 to 3.3)	<0.0001	2.6 (1.8 to 3.7)	<0.0001
CNS Tumors							
Astrocytoma	52 (2.1%)	930 (13.9%)	5.3	REF			
Ependymoma	38 (1.5%)	159 (2.4%)	19.3	4.3 (2.7 to 6.7)	<0.0001	4.5 (2.7 to 7.3)	<0.0001
Medulloblastoma	36 (1.4%)	136 (2.0%)	20.9	4.7 (3.0 to 7.5)	<0.0001	4.6 (2.8 to 7.6)	<0.0001
Other brain tumor	111 (4.4%)	845 (12.7%)	11.6	2.3 (1.7 to 3.3)	<0.0001	2.6 (1.8 to 3.7)	<0.0001
Socioeconomic Factors							
Median Km to Nearest Tertiary Care Center (Interquartile Range)	28.0 (12.4 to 99.4)	30.9 (12.0 to 118.2)		Per 100 km 0.96 (0.94 to 0.98)	<0.0001	Per 100 km 0.96 (0.94 to 0.98)	0.0005
Income quintile					0.523		0.908
1 (lowest)	452 (17.8%)	1280 (19.2%)	26.1	REF		REF	
2	489 (19.3%)	1270 (19.0%)	27.8	1.1 (0.9 to 1.3)	0.257	1.1 (0.9 to 1.3)	0.445
3	508 (20.1%)	1306 (19.6%)	28.0	1.1 (1.0 to 1.3)	0.201	1.0 (0.9 to 1.2)	0.718
4	522 (20.6%)	1321 (19.8%)	28.3	1.1 (1.0 to 1.3)	0.135	1.0 (0.9 to 1.2)	0.861
5 (highest)	539 (21.3%)	1354 (20.3%)	28.5	1.1 (1.0 to 1.3)	0.109	1.0 (0.8 to 1.2)	0.900

*P value by logistic regression

*Abbreviations*: *OR* odds ratio, *CI* confidence interval, *REF* reference

We then conducted the subgroup analysis in which multiple regression was repeated after removal of patients in whom non-enrollment reason was stated as “No available trial”. Asian and Arab/west Asian race remained significantly associated with lower enrollment on trials with OR 0.6 (95% CI 0.4 to 0.97; *P* = 0.037) and OR 0.4 (95% CI 0.2 to 0.8; *P* = 0.004) respectively. Children with ALL had a 26 fold increased odds of trial enrollment compared to children with astrocytoma in this subgroup analysis (OR 26.2, 95% CI 16.8 to 40.7; *P* < 0.0001).

## Discussion

In this population based study, we found that the proportion of pediatric cancer patients enrolled on a therapeutic trial for the initial diagnosis of cancer was 27.5%. We found that when the reason for non-enrollment was documented, the two most common were lack of trial availability and physician choice. Factors associated with lower enrollment were Asian and Arab/west Asian race, underlying diagnosis and greater distance from the nearest tertiary care hospital.

In trying to place our findings in context to the existing literature, it becomes clear that other studies reporting the number of children enrolled to trials differed in their methodology and approach, with many reporting that more children were enrolled on trials compared with our study. For example, a report from the United Kingdom Children’s Cancer Study Group noted that 70% of all children were enrolled to trials. However, it was not clear whether the denominator reflected children diagnosed when a study was open for accrual, those who were eligible when a study was available, or all children diagnosed with cancer [10]. A report concluding that 86.1% of patients ≤21 years of age were enrolled on any trial excluded patients who were diagnosed when a trial was not available [5]. Another study described that 73% of potentially *eligible* patients with leukemia were enrolled on a trial with the assumption that a trial was available throughout the time frame of evaluation [11].

When studies evaluated a similar metric to ours, namely the proportion of all children diagnosed with cancer enrolled on a therapeutic clinical trial, similar findings were observed. For example, Dodgshun et al. found that among children ≤16 years of age with cancer diagnosed between 2009 and 2010 in New Zealand, 27.5% were enrolled on a trial [12]. Single center studies from Seattle [13] and Pittsburgh [4] demonstrated that 48.8% and 38% were enrolled on therapeutic trials.

We found that the two most common reasons cited for non-enrollment were lack of trial availability and physician decision. Unfortunately, we do not know from the data whether lack of trial availability reflected lack of any trial for that diagnosis, or whether a trial was available but not activated at the center because of regulatory/ethics barriers or delays, or whether the center had chosen not to open an available study. This finding suggests that if trial enrollment is important, barriers to having trials available should be identified and mechanisms to facilitate and expedite trial activation should be implemented. The high proportion of physicians deciding to not enroll children on trials is interesting and should be explored further to better understand reasons for not offering eligible children a clinical trial. It was also surprising and encouraging that patient refusal was an uncommon reason for non-participation. However, this figure does not imply that patients understand the consenting process or that they are later satisfied with their decision. These are important research questions.

Risk factors for non-enrollment previously identified have been older age [5, 14, 15], diagnosis of lymphoma and brain tumor [12] and Hispanic patients or those with Spanish-speaking parents [5]. Interestingly, our study had different findings with age not being a significant factor in the adjusted model, which may be the result of most children with ALL being younger. However, we found Asian race was negatively associated with trial enrollment, which is a novel finding. The association between underlying diagnosis and non-enrollment does not solely reflect the availability of clinical trials as shown by our subgroup analysis in which we removed patients in whom lack of trial availability was stated as the reason for non-enrollment.

The strengths of this study were its population-based nature and the consistent collection of reasons for non-enrollment from the 12 non-Ontario centers. However, these results must be interpreted in light of its limitations. First, we did not describe outcomes of patients enrolled and not enrolled on clinical trials. We felt that describing outcomes was outside the scope of this paper as each diagnosis will need to be addressed separately since consideration of confounding variables and risk stratification will differ by diagnosis type. Second, we only evaluated clinical trial enrollment at initial cancer diagnosis and not at refractory disease or relapse, another important future research gap. Third, an important limitation of our study is that we did not include adolescent and young adult patients (AYA) in our study. This is important as several studies have identified that AYA patients have lower rates of enrollment on clinical trials. [5, 14, 15] Our study suggests that in general, younger patients have poor rates of enrollment to trials; this implies that AYA enrollments may even be worse than previously thought when evaluated on a population basis. Fourth, an important limitation is that we excluded more than 10% of the sample because of unknown trial status. This exclusion may mean that our rates of enrollment are falsely high and is an important fact to stress when citing these results. Also, there was a high rate of missing reasons for failure to enroll on a trial, thus meaning that factors less likely to be recorded in the medical records, such as physician preference, may be under-represented. However, factors such as patient refusal should have been well documented and are thus unlikely to be biased. Finally, we found that those with missing enrollment information were significantly different by age and diagnosis, thus potentially limiting the generalizability of our findings (data not shown).

## Conclusions

In conclusion, in Canada, 27.5% of children with cancer are enrolled onto therapeutic clinical trials and lack of an available trial is the most common stated reason contributing to non-enrollment. Future research should better understand reasons for lack of trial availability and physician preferences to not offer trials.

## Abbreviations

ALL: Acute Lymphoblastic Leukemia
AYA: Adolescent and Young Adult
CYP-C: Cancer in Young People in Canada
ICCC: International Classification of Childhood Cancer
OR: Odds Ratio
POGO: Pediatric Oncology Group of Ontario
POGONIS: Pediatric Oncology Group of Ontario Networked Information System
